# Mapping Theme Trends and Knowledge Structure of Magnetic Resonance Imaging Studies of Schizophrenia: A Bibliometric Analysis From 2004 to 2018

**DOI:** 10.3389/fpsyt.2020.00027

**Published:** 2020-02-07

**Authors:** Li Duan, Gang Zhu

**Affiliations:** ^1^Department of Psychiatry, the First Affiliated Hospital of China Medical University, Shenyang, China; ^2^Central Laboratory, the First Affiliated Hospital of China Medical University, Shenyang, China

**Keywords:** schizophrenia, magnetic resonance imaging, bibliometric analysis, co-occurrence analysis, social network analysis, strategic diagram

## Abstract

**Background:**

Recently, magnetic resonance imaging (MRI) technology has been widely used to quantitatively analyze brain structure, morphology, and functional activities, as well as to clarify the neuropathological and neurobiological mechanisms of schizophrenia. However, although there have been many relevant results and conclusions, there has been no systematic assessment of this field.

**Aim:**

To analyze important areas of research utilizing MRI in studies of schizophrenia and explore major trends and the knowledge structure using bibliometric analysis.

**Methods:**

Literature related to MRI studies of schizophrenia published in PubMed between January 1, 2004 and December 31, 2018 were retrieved in 5-year increments. The extracted major Medical Subject Headings (MeSH) terms/MeSH subheadings were analyzed quantitatively. Bi-clu-stering analysis, social network analysis (SNA), and strategic diagrams were employed to analyze the word matrix and co-occurrence matrix of high-frequency MeSH terms.

**Results:**

For the periods of 2004 to 2008, 2009 to 2013, and 2014 to 2018, the number of relevant retrieved publications were 916, 1,344, and 1,512 respectively, showing an overall growth trend. 26, 34, and 36 high-frequency major MeSH terms/MeSH subheadings were extracted in each period, respectively. In line with strategic diagrams, the main undeveloped theme clusters in 2004–2008 were effects of antipsychotics on brain structure and their curative efficacy. These themes were replaced in 2009–2013 by physiopathology mechanisms of schizophrenia, etiology of cognitive disorder, research on default mode network and schizophrenic psychology, and were partially replaced in 2014–2018 by studies of differences in the neurobiological basis for schizophrenia and other mental disorders. Based on SNA, nerve net/physiopathology and psychotic disorder/pathology were considered the emerging hotspots of research in 2009–2013 and 2014–2018.

**Conclusions:**

MRI studies on schizophrenia were relatively diverse, but the theme clusters derived from each period may reflect the publication trends to some extent. Bibliometric research over a 15-year period may be helpful in depicting the overall scope of research interest and may generate novel ideas for researchers initiating new projects.

## Introduction

Schizophrenia, as a common and devastating mental disorder, has been characterized by abnormal social behavior and the failure to understand reality, accompanied by emotional disorder or substance abuse, and even suicide (approximately 5%) (1), which leads to high risk for physical diseases, increased disability and recurrence for patients, and places a serious burden on their families and society (2, 3). The prevalence of schizophrenia has been stable at around 1% (4), and the years lived with disability (YLD) due to schizophrenia in 2016 was estimated to be 13.4 million years [95% uncertainty interval (UI), 9.9–16.7 million years], accounting for 1.7% of all YLDs and ranking 15th among all diseases worldwide (5). Nevertheless, the underlying etiology and pathogenesis of schizophrenia have not been fully elucidated, but scholars have come to the consensus that it is a disabling encephalopathy caused by a group of genetic factors, as well as heterogeneous neurodevelopmental risk factors and adverse environmental stimuli (6–9).

At present, clinical diagnosis of schizophrenia is mainly based on patients’ abnormal behavior. Clinical symptoms are described with quantitative dimensions, such as the quantitative evaluation of symptoms with the Positive and Negative Syndrome Scale (10). However, when patients exhibit typical psychotic symptoms and abnormal behaviors, they usually have advanced or relapsed into the middle and late stages of schizophrenia, which is not conducive to the implementation of rapid and effective treatment programs and the long-term recovery of patients. In recent years, neuroimaging technology has become an important way to understand normal physiological functions and pathological phenomena in the brain. Although patients with schizophrenia usually have anomalous brain structure and function, as well as neurological damage, it is possible for psychiatrists to explore early diagnosis, screen for specific biomarkers, monitor pathogenetic progression and therapeutic efficacy, and uncover pathophysiological mechanisms by employing structural and functional imaging technologies (11–15). Magnetic resonance imaging (MRI) scanning techniques focus primarily on structural magnetic resonance imaging, resting-state functional magnetic resonance imaging and diffuse tensor imaging (DTI). As early as 1927, scientists began to perform brain scans of schizophrenia patients, but the traumatic operation (injecting air into the cerebrospinal canal) meant that patients had to endure pain for months (16). It wasn’t until 1976 that Johnstone used computerized tomography technology to make the first non-invasive study of the brain of schizophrenia patients with enlarged lateral ventricles (17). Later, to improve the poor discrimination of soft tissue, Smith et al. conducted the first MRI study on schizophrenia patients in 1984 (18). Depending on the characteristics of the various imaging diagnostic techniques employed, the scope of MRI applications may differ; for example, structural magnetic resonance imaging (sMRI) enables high resolution spatial imaging of the brain *in vivo* based on its unique advantages which provide noninvasive, harmless and high spatial resolution, while DTI allows observation of the direction and structural integrity of nerve fiber bundles in the white matter *in vivo*, and functional MRI (fMRI) can detect functional activity of the brain. Furthermore, with the development of magnetic resonance technology, researchers are gradually extending their exploration of the brain of patients with schizophrenia and achieving fruitful results to better carry out clinical work and provide more professional advice for patients.

Bibliometrics, as a branch of library and information science, originated in the early 20^th^ century. It is a comprehensive application of mathematics and statistics to conduct quantitative analysis and description of various characteristics of published literatures, so as to provide an easy method to evaluate research status and predict the developmental trends (19). Therefore, bibliometric analysis, including co-citation analysis, co-word analysis, and other methods, can help researchers grasp the changing trends of a given research field (20). While these methods have enriched the research content of MRI studies on schizophrenia, at present, few studies have attempted to focus on and sort out the important issues and research context from the perspective of bibliometrics. In view of this situation, this study attempted to analyze and summarize the internal and external characteristics of related literatures and track and intuitively display the research trends and frontiers in the field of MRI studies on schizophrenia by the integrated application of co-word analysis, bi-clustering analysis, strategic diagram, and social network analysis (SNA). Our goal is to promote public knowledge and provide reference for the increasing depth and breadth of future research on schizophrenia.

## Methods

### Data Collection, Data Extraction, and Bibliographic Matrix Setup

Literature involved in this study were retrieved and downloaded from the PubMed database(National Center for Biotechnology Information, U.S. National Library of Medicine, Rockville Pike, Bethesda MD, USA). Medical Subject Headings (MeSH) which are characterized by accuracy (accurately revealing the subject of the literature) and specificity can be used to index and catalog literatures in PubMed. In this study, the retrieval model was set as [“Magnetic Resonance Imaging”(Mesh) OR “Diffusion Tensor Imaging”(Mesh) OR “Diffusion Tensor Imaging”(Title/Abstract) AND “Schizophrenia”(Mesh)] with the filter restriction of literature type as “journal article” and language as “English”. In addition, in order to dynamically analyze the changes in hotspots, theme trends and knowledge structure of related studies of MRI and schizophrenia, the publication scope was divided into three periods (January 1, 2004 to December 31, 2008, January 1, 2009 to December 31, 2013, and January 1, 2014 to December 31, 2018). Finally, 916, 1,344, and 1,512 related literatures were retrieved in each period, respectively. Additionally, two researchers are required to carry out the primary retrieval and literature screening based on reviewing titles, abstracts, as well as full text in some cases independently.

Bibliographic information, including publication dates, countries, titles, authors, journal categories, major MeSH terms, MeSH subheading terms, abstracts, and other related characteristics of these literatures were accurately extracted and properly kept in XML format. The principle of h-index (21) was followed to set the threshold value of the high- and low-frequency major MeSH terms/MeSH subheadings. As a hybrid quantitative index, it was originally proposed by Hirsch to quantify the output of an individual researcher and gradually expand the index to evaluate the influence of patents (22), academic journals (23), research institutions (24), and so on. In this study, the Bibliographic Item Co-occurrence Matrix Builder (BICOMB) (25) was applied to read and analyze the bibliographic information of these retrieved literatures. Word frequency statistics in descending order were obtained with “major MeSH terms/MeSH subheadings.” When the word frequency was consistent with its rank, all major MeSH terms/MeSH subheadings greater than or equal to the rank were considered high frequency. Thereafter, fundamental data from the term-source literature and the term-term co-occurrence matrix were also generated to prepare for the subsequent bibliometric analysis.

### Bi-Clustering Analysis of High-Frequency Mesh Terms

Bi-clustering analysis, also known as two-way clustering, was first proposed by Hartigan in 1972 (26). It refers to the concurrent clustering of rows and columns of data, and the simultaneous application of objects and their attributes to extract their common information. In this study, bi-clustering analysis of high-frequency major MeSH terms/MeSH subheadings was conducted on the basis of the term-source literature matrix by employing gCLUTO (Graphical Clustering Toolkit) software (http://glaros.dtc.umn.edu/gkhome/cluto/gcluto/download), with the aim of assessing the knowledge structure of studies on MRI of schizophrenia. The results are presented in the form of a visual matrix and visual mountain.

The two-dimensional matrix visualization, as a colorful interactive matrix, is composed of horizontal rows of high-frequency major MeSH terms/MeSH subheadings and vertical columns of PubMed unique identifiers (PMID) of these retrieved literatures, which are showed on the left and the top of the matrix, respectively. Mountain visualization attempts to describe the relationship between clusters from a three-dimensional perspective, and we can estimate the relative similarity between peaks by estimating the distance between them. The volume and height of each mountain is proportional to the number of high-frequency MeSH terms contained in a cluster and their similarity within the cluster, respectively. The greater the similarity within a cluster, the steeper the mountain. In addition, the colors of peaks include red, yellow, light blue, and dark blue. Red represents a lower standard deviation of the internal similarity in the cluster, while blue denotes a higher standard deviation. In addition, the closer the color is to a single color, the smaller is the deviation between the internal MeSH terms of various clusters and the greater is their similarity.

Furthermore, according to calculate statistical indices, including descriptive (literatures that represents this class of characteristics) and discriminating (literatures that distinguishes it from other clusters) of each high-frequency major MeSH terms/MeSH subheadings to the clusters, trace back to the source literatures by identifying the PMID that contributes the most to the formation of each cluster as the significant representative literatures. These extracted representative literatures can be used to summarize and interpret content of the theme cluster.

### Strategic Diagram Analysis

The analysis method of strategic diagram was first proposed by Law et al. in 1988 and aimed to describe the complex internal structure and developmental trend of hotspots in a research field on the basis of the co-occurrence matrix and bi-cluster analysis (27). The two-dimensional strategic diagram (28), formed by employing software of GraphPad Prism 5.0 (Graphpad, Inc., La Jolla, CA, USA), places on the horizontal axis centrality or degree of external cohesion, namely, the central position of the theme cluster with other clusters. The vertical axis specifies the density or degree of internal cohesion, namely, the conceptual development of the theme cluster.

The strategic diagram can be divided into four quadrants moving counterclockwise (**Figure 5A**). The theme cluster which is located in the first quadrant (Quadrant I, upper right), represents the development core and relatively mature hotspots in the field, implying strong centrality, and high density. Theme clusters in the second quadrant (Quadrant II, upper left) are characterized by inadequate external interactions, but high density of terms in periphery development in this research field. The third quadrant (Quadrant III, lower left) consists of theme clusters with weak centrality and low density, which are identified as emerging or vanishing. Although the density of theme clusters contained in the fourth quadrant (Quadrant IV, lower right) is low, it has high centrality, which indicates that its internal structure is loose and its development has not achieved sufficient maturity.

### Social Network Analysis

SNA, as an increasingly applied and rapidly developing method in variety of fields (e.g., psychology, sociology, mathematics, statistics, and others), emphasizes the connectivity and interdependence of elements in a group (29). In this study, to analyze and interpret the theme trends and knowledge structural characteristics of MRI studies of schizophrenia, we employed the “centrality” concept from SNA on the basis of the high-frequency major Mesh terms/MeSH subheadings co-occurrence matrix. Furthermore, to assess the importance of each node, statistical indices including degree, betweenness, and closeness centrality were selected to perform the network analysis. The connotation of each index is explained as follows: (1) Degree centrality is calculated by the number of direct links associated with or connected to one node within the network (30). This index is suitable for evaluating the co-occurrence level among nodes, indicating the importance of one given node to the network. This is evaluated by their degree centrality. (2) Betweenness centrality is calculated by the number of shortest paths between two other nodes that pass through one given node (31). This index can indicate the influence of this node to the network, that is, the higher the betweenness centrality value, the more powerful is this node in controlling other nodes within a network (32). (3) Closeness centrality also can be used as another measure for quantifying the importance of a given node (33). In contrast to betweenness centrality, this index is calculated from the reciprocal of the sum of the lengths of the shortest paths between the node and all other nodes within the network. Therefore, the larger the closeness centrality value of the given node, the closer it is to all the other nodes within the network.

Given that betweenness centrality, as a mediating role, is more applicable to describe the decisive effect within the whole network, we chose it to scale the node sizes. Calculation of the related statistical indices and drawing of the network diagrams were completed using Ucinet 6.0 software (Analytic Technologies Co., Nicholasville, Kentucky, USA), while visualization of the network structure was demonstrated using NetDraw 2.084 software (http://www.analytictech.com/downloadnd.htm).

## Results

### Distribution Characteristics of Related Publications

**Figure 1:**
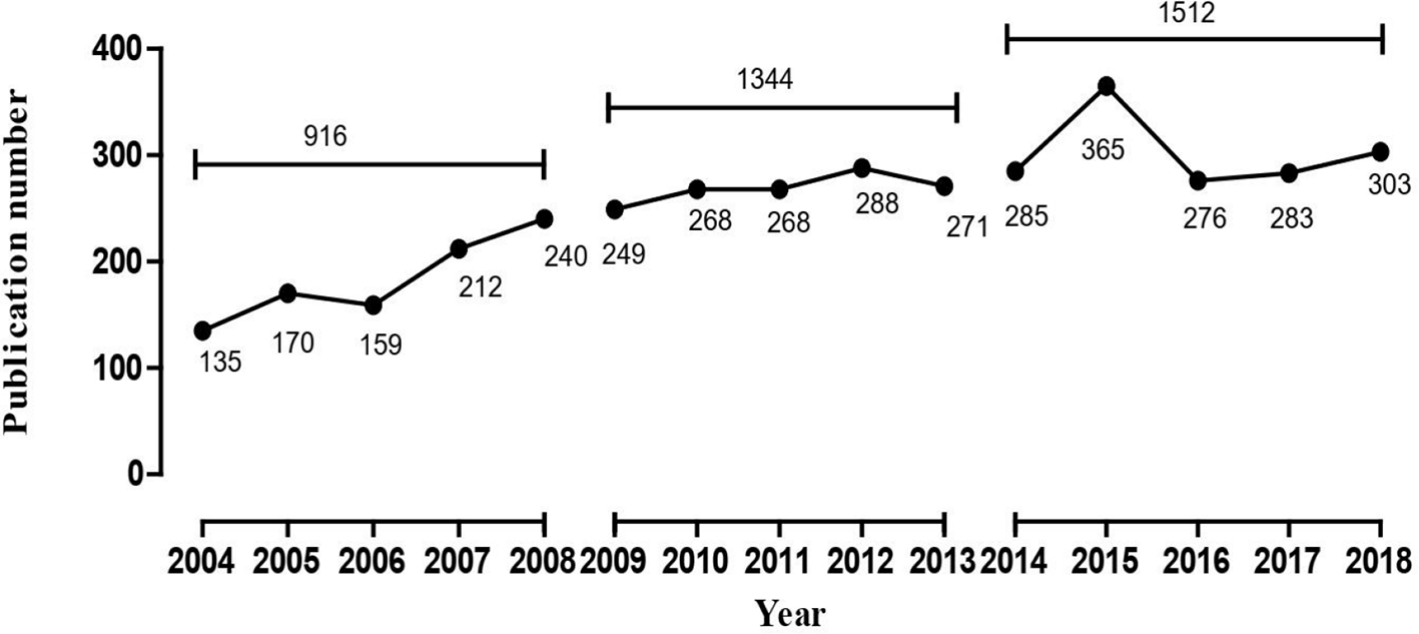
The number of publications of MRI studies on schizophrenia in PubMed from 2004 to 2018.

In this study, 916, 1,344, and 1,512 literatures were retrieved from the three periods of 2004 to 2008, 2009 to 2013, and 2014 to 2018, respectively, which were then subjected to comparative analysis using the statistical indices of authors, source of countries, and journals. The annual total number of MRI studies on schizophrenia presented an overall growth trend (**Figure 1**). The top five countries in this research field in the first period, in descending order of production, were the United States, England, Netherlands, Ireland, and Germany, while Netherlands overtook England for the second place in other two periods (**Table 1**). Additionally, it was clear that the proportion of publications in the U.S., which was the highest in all three periods, has been trending down, while Netherlands and England’s percentage of publication have continuously increased. The top two journals were *Schizophrenia Research* and *Psychiatry Research* in the first two periods, while *Biological Psychiatry* in 2004–2013 was replace by *Schizophrenia Bulletin* in 2009–2013. These three journals together contained more than 33.54% and 33.65% of the total number of searched publications in this field in the first two periods, respectively. From 2014 to 2018, the top three journals were *Schizophrenia Research*, *Schizophrenia Bulletin*, and *NeuroImage. Clinical*. Furthermore, the journal *Schizophrenia Research* ranked first in the number of articles published in all three periods, but its proportion of the total number of searched publications declined slightly. In addition, Shenton ME was the greatest contributor to MRI studies on schizophrenia research in the first period, while Calhoun VD contributed the most academic papers in the last two periods.

**Table 1:** Temporal distribution of publications of MRI studies on schizophrenia in PubMed from 2004 to 2018.

Period	Rank	Country		Top journal		Author	
		Name	Publications, n (%)	Title	Publication, n (%)	Name	Number of papers
2004- 2008	1	United States	427(44.11%)	*Schizophrenia research*	162(16.71%)	Shenton ME	35
	2	England	178(18.39%)	*Psychiatry research*	86(8.88%)	McCarley RW	34
	3	Netherlands	178(18.39%)	*Biological psychiatry*	77(7.95%)	Lawrie SM	30
	4	Ireland	96(9.92%)	*NeuroImage*	73(7.53%)	Johnstone EC	29
	5	Germany	42(4.34%)	*The American journal of psychiatry*	67(6.91%)	Keshavan MS	26
	Total		921(95.15%)		465(47.98%)		
2009- 2013	1	United States	574(39.70%)	*Schizophrenia research*	242(16.69%)	Calhoun VD	58
	2	Netherlands	285(19.71%)	*Psychiatry research*	132(9.10%)	Shenton ME	41
	3	England	273(18.88%)	*Schizophrenia bulletin*	114(7.86%)	Keshavan MS	40
	4	Ireland	144(9.96%)	*NeuroImage*	96(6.62%)	Kubicki M	37
	5	Germany	70(4.84%)	*Biological psychiatry*	61(4.20%)	Kahn RS	35
	Total		1346(93.09%)		645(44.47%)		
2014- 2018	1	United States	573(37.26%)	*Schizophrenia research*	222(14.37%)	Calhoun VD	77
	2	Netherlands	425(27.63%)	*Schizophrenia bulletin*	125(8.09%)	Pearlson GD	36
	3	England	311(20.22%)	*NeuroImage. Clinical*	81(5.24%)	Andreassen OA	36
	4	Ireland	74(4.81%)	*Psychiatry research. Neuroimaging*	63(4.08%)	Agartz I	35
	5	Germany	45(2.93%)	*Psychiatry research*	62(4.01%)	Guo W	34
	Total		1428(92.85%)		553(35.79%)		

### Research Hotspots Identified and Theme Clusters Summarized Based on MeSH Term Clusters

From the searched literature, 26, 34, and 36 high-frequency major MeSH terms/MeSH subheadings were extracted in each period, respectively, and their cumulative frequency percentages were 49.0459, 53.8805, and 52.1285% of the total, and thus could be considered as the research hotspots of MRI studies on schizophrenia in the past three 5-year time periods (**Table 2**). And, it should be pointed out that in the second period of 2009–2013, the word frequency values of 33rd and 34th major MeSH terms/MeSH subheadings were the same as 33, so we extracted 34 high-frequency MeSH terms.

**Table 2:** Distribution of the high-frequency major MeSH terms/MeSH subheadings of MRI studies on schizophrenia in PubMed from 2004 to 2018.

Period	Threshold value of high- and low-frequency of MeSH terms	Number of high-frequency of MeSH terms	Frequency		Cumulative frequency
			Min	Max	
2004-2008	26	26	26	327	49.0459%
2009-2013	33	34	33	663	53.8805%
2014-2018	36	36	36	605	52.1285%

**Figure 2:**
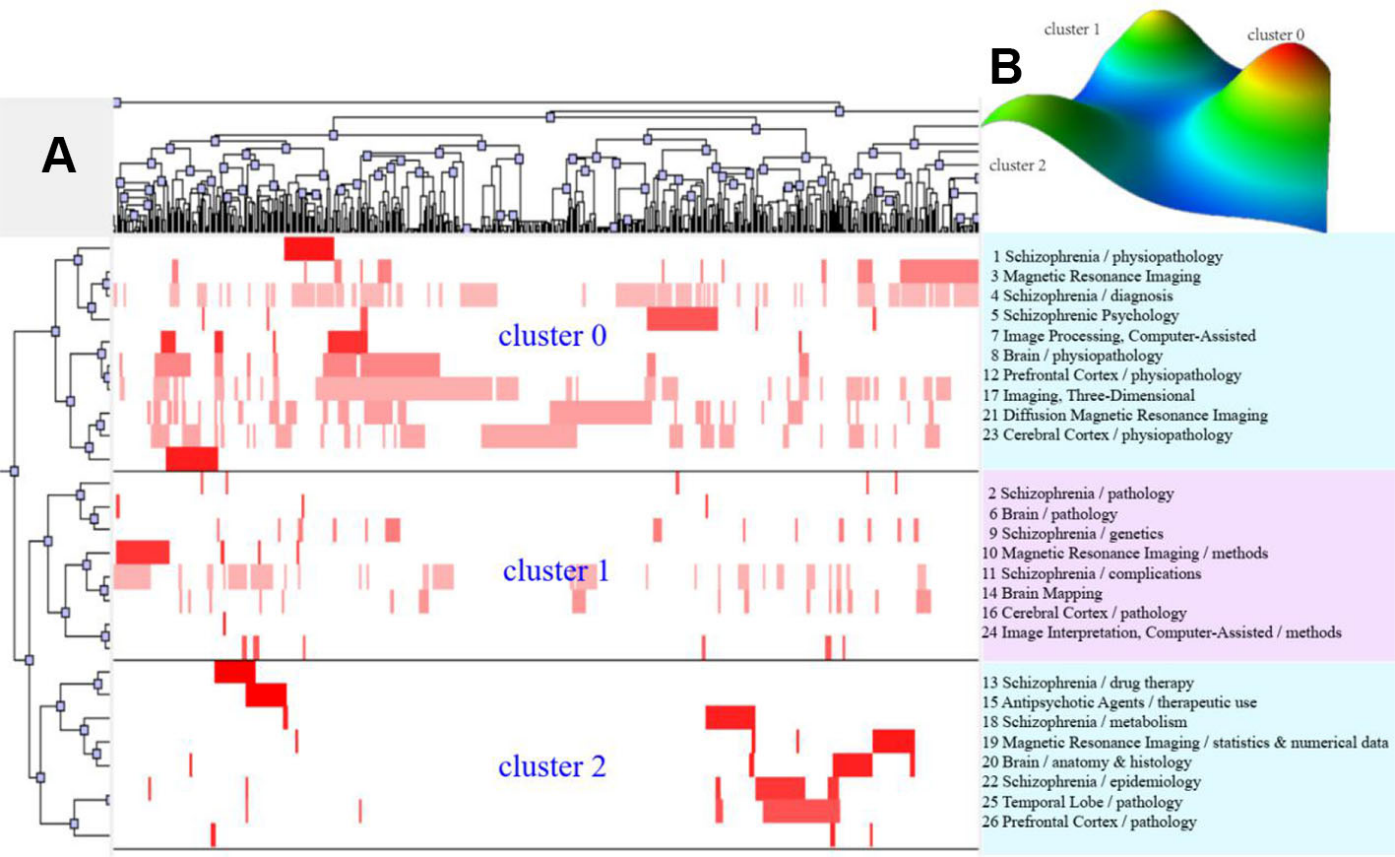
Bi-clustering analysis of 26 high-frequency MeSH terms/MeSH subheadings and literatures of MRI studies on schizophrenia in 2004 to 2008. **(A)** Matrix visualization of bi-clustering of 26 high-frequency major MeSH terms/MeSH subheadings and PubMed unique identifiers of literatures. **(B)** Mountain visualization of bi-clustering of 26 high-frequency major MeSH terms/MeSH subheadings and literatures.

**Figure 3:**
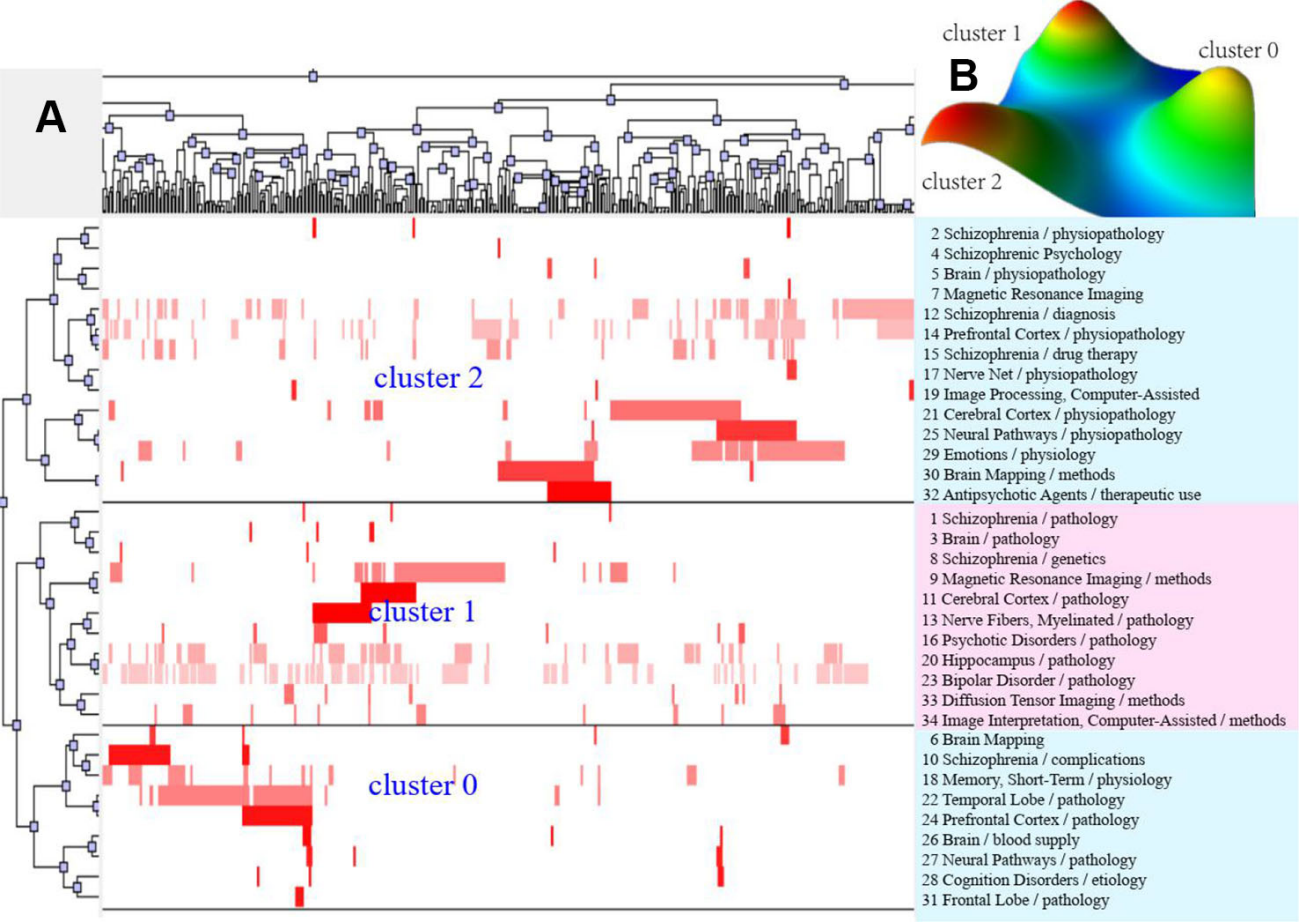
Bi-clustering analysis of 34 high-frequency major MeSH terms/MeSH subheadings and literatures of MRI studies on schizophrenia in 2009 to 2013. **(A)** Matrix visualization of bi-clustering of 34 high-frequency major MeSH terms/MeSH subheadings and PubMed unique identifiers of literatures. **(B)** Mountain visualization of bi-clustering of 34 high-frequency major MeSH terms/MeSH subheadings and literatures.

**Figure 4:**
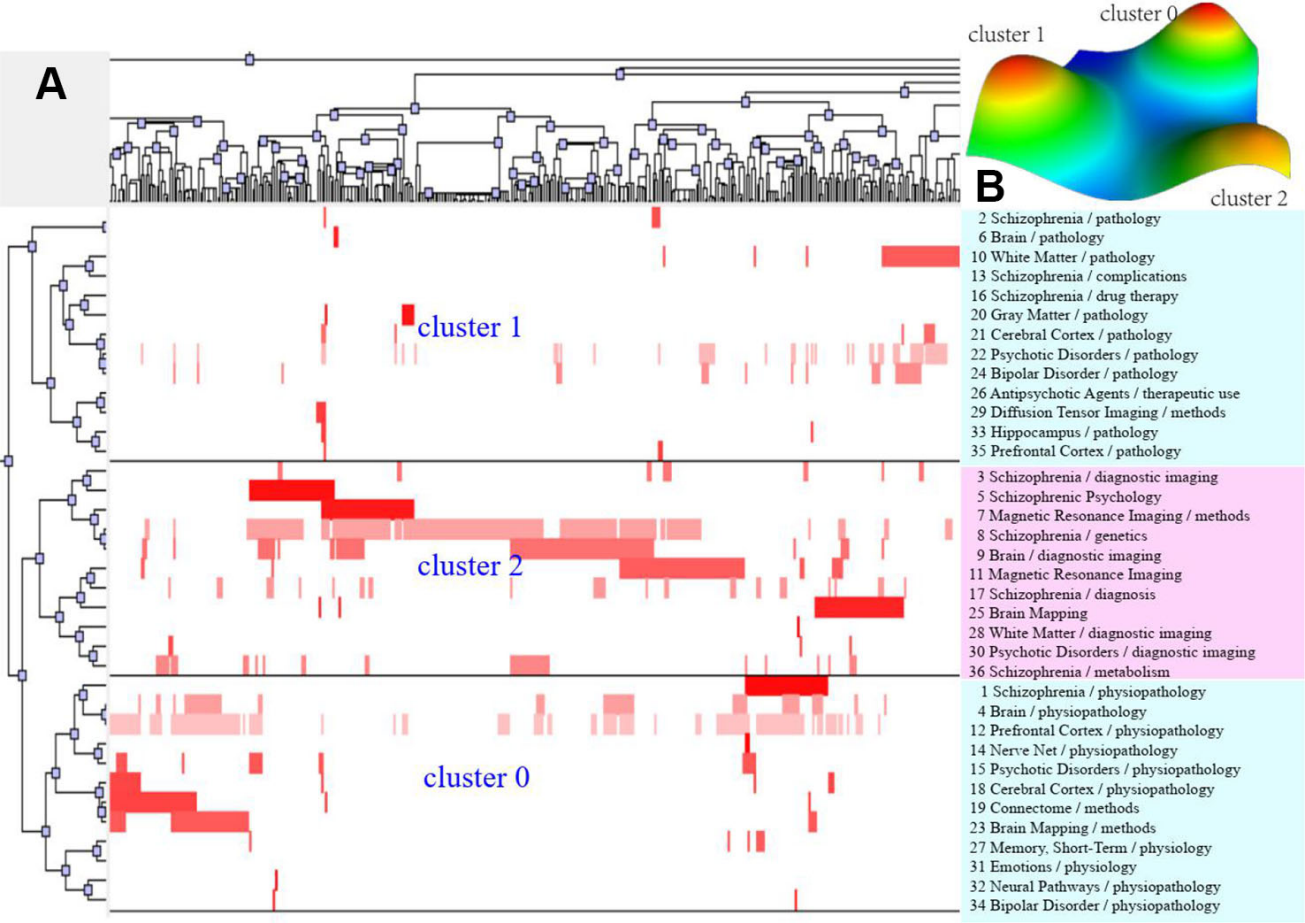
Bi-clustering analysis of 36 high-frequency major MeSH terms/MeSH subheadings and literatures of MRI studies on schizophrenia in 2014 to 2018. **(A)** Matrix visualization of bi-clustering of 36 high-frequency major MeSH terms/MeSH subheadings and PubMed unique identifier of literatures. **(B)** Mountain visualization of bi-clustering of 36 high-frequency major MeSH terms/MeSH subheadings and literatures.

The high-frequency MeSH terms in each period can be seen in **Figures 2**–**4**.

According to bi-clustering analysis, 26, 34, and 36 high-frequency major MeSH terms/MeSH subheadings in each period were evenly divided into three clusters (**Figures 2**–**4**). For the results of mountain visualization, Cluster 0 in the first period, Clusters 1 and 2 in the second period, and Clusters 0 and 1 in the third period were marked with red peaks, which represented the most significant results with centralized distribution and low internal standard deviation of the internal similarity within clusters. Furthermore, we have made explicit the relationships among high-frequency major MeSH terms/MeSH subheadings and involved literatures from matrix visualization in each period. The major MeSH terms/MeSH subheadings contained in each cluster are displayed on the right side of the matrix, and the number before them referred to the descending rank of the frequency or proportion of frequency from the included literatures in each period. Moreover, an in-depth understanding of the meaning of the MeSH terms themselves, term-source literatures, as well as the significant representative literatures selected by bi-clustering analysis would be conducive to analyze and summarize the theme of each cluster. At the same time, it would also helpful to further interpret the connotation of the relevant literatures in each cluster.

### Theme Trends of MRI Studies on Schizophrenia

**Figure 5:**
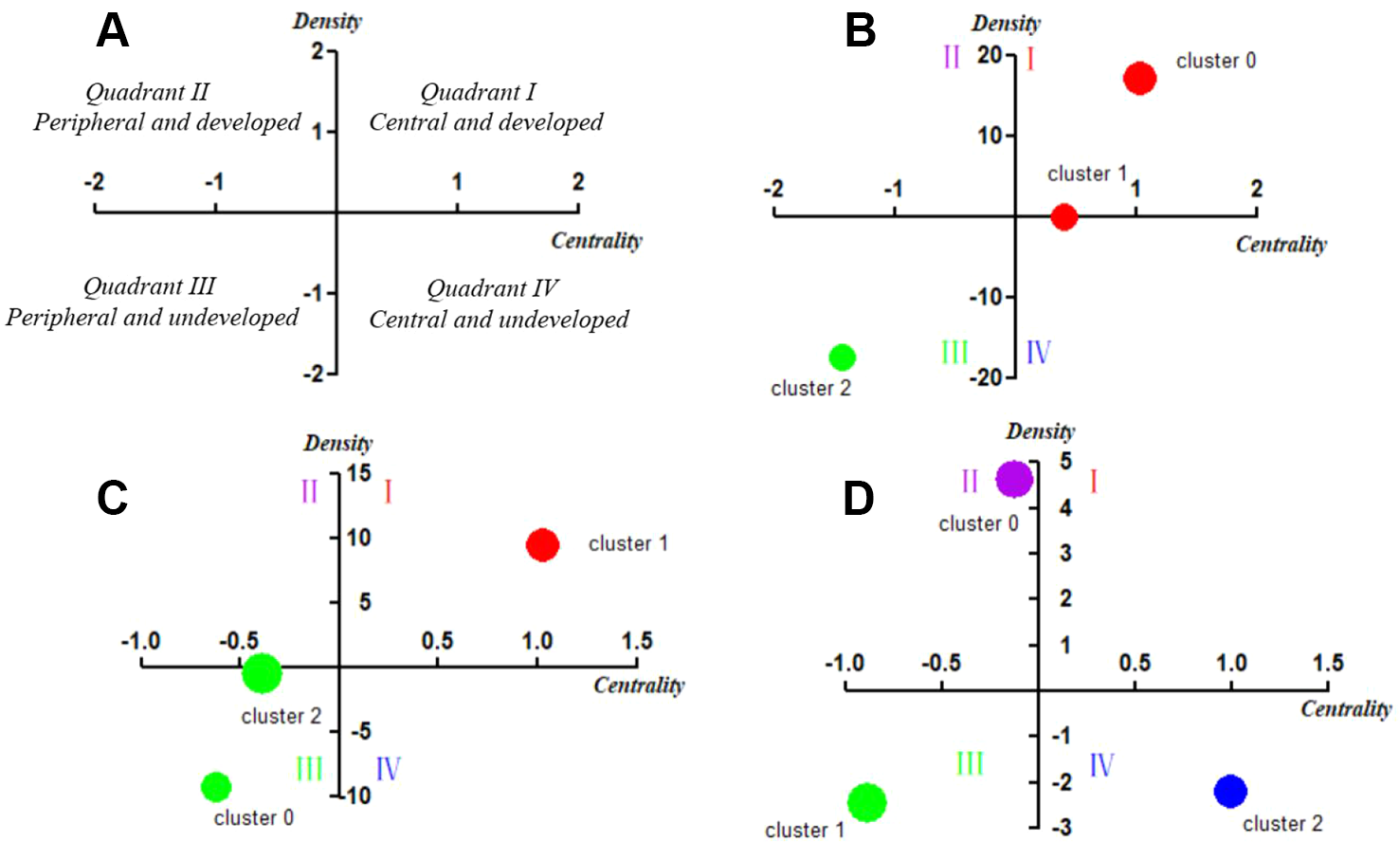
Strategic diagrams for MRI studies on schizophrenia in three different periods. **(A)** Explanation of the strategic diagram. **(B)** Strategic diagram for MRI studies on schizophrenia in 2004–2008. The coordinate values of Cluster 1 are (0.40787, 0.07288), which locates it in Quadrant I. **(C)** Strategic diagram for MRI studies on schizophrenia in 2009–2013. **(D)** Strategic diagram for MRI studies on schizophrenia in 2014–2018. The coordinate values of Cluster 2 are (-0.39885, -0.37111), which locates it in Quadrant I. Clusters in each strategic diagram refer to the bi-clustering results presented in **Table 3**. The size of each single node is proportional to the number of high-frequency major MeSH terms/MeSH subheadings involved in each cluster (**Figures 5 A – D**).

In this study, we plotted strategic diagrams so as to systematically compare similarities and differences of the theme clusters in the three periods and explore the developmental trends of MRI studies on schizophrenia. The number of high-frequency major MeSH terms/MeSH subheadings involved in each cluster can be reflected by the area of the nodes, that is, the greater the number, the larger the area (**Figure 5B–D**).

**Table 3:** Cluster interpretation of high-frequency major MeSH terms/MeSH subheadings of MRI studies on schizophrenia in PubMed from 2004 to 2018.

Period	Cluster	Content and interpretation of the cluster	Rank of MeSH terms	Representative literatures (PMID)
2004-2008	Cluster 0	Correlation between brain structural (e.g. cerebral cortex, prefrontal cortex) abnormalities and brain function changes to explore physiopathology mechanism of schizophrenia	1, 3, 4, 5, 7, 8, 12, 17, 21, 23	17085018, 17913465, 16797186, 19042913
	Cluster 1	1. Neurodevelopmental abnormalities in patients with schizophrenia	2, 6, 9, 10, 11, 14, 16, 24	18793730, 17166743, 17689500, 16179202
		2. Analysis of regional abnormal structural features and influencing factors of the brain		
	Cluster 2	1. Analysis of metabolism, therapeutic efficacy of antipsychotic agents on brain structure	13, 15, 18, 19, 20, 22, 25, 26	15201569, 17507880, 15520356, 16005383, 15809403
		2. Schizophrenia pathology (from the perspective of temporal lobe, prefrontal cortex and anatomy and histology of brain structure)		
2009–2013	Cluster 0	Research on the physiopathology mechanism (from the perspective of frontal lobe, temporal lobe, prefrontal cortex, neural pathways) of schizophrenia and the etiology of cognitive disorder and other complications	6, 10, 18, 22, 24, 26, 27, 28, 31	19097861, 20452574, 21868203, 19683896, 22105156
	Cluster 1	1. Research on brain structure and function (e.g. hippocampus, nerve fibers, cerebral cortex) and differentiate schizophrenia from bipolar disorder based on MRI	1, 3, 8, 9, 11, 13, 16, 20, 23, 33, 34	24004694, 22832855, 20573561, 21878411, 19042913, 22945617
		2. MRI (including DTI) usages, image interpretations		
	Cluster 2	1. Research on default mode network in schizophrenia patients	2, 4, 5, 7, 12, 14, 15, 17, 19, 21, 25, 29, 30, 32	21095105, 21147518, 19931396, 21277171
		2. Schizophrenic psychology (association between anatomical and functional cerebral deficits with related clinical symptoms)		
2014–2018	Cluster 0	Research on brain structural and functional abnormality (e.g. prefrontal cortex, nerve net) of patients with schizophrenia, as well as physiopathology mechanism of the disease	1, 4, 12, 14, 15, 18, 19, 23, 27, 31, 32, 34	24306091, 26123450, 28338738, 29527474, 26908926, 28207073
	Cluster 1	1. Research on the difference of neurobiological basis between schizophrenia and other mental diseases	2, 6, 10, 13, 16, 20, 21, 22, 24, 26, 29, 33, 35	28347393, 25904725, 25089761, 29257977, 25829144, 25968549
		2. Effects of antipsychotics on brain structural changes		
	Cluster 2	1. Effects of genetic load on brain function of schizophrenia patients	3, 5, 7, 8, 9, 11, 17, 25, 28, 30, 36	27479923, 27375133, 27829096, 29935206, 29247760
		2. Identification of schizophrenia biomarkers		

“Rank of MeSH terms” represents the number of high-frequency major MeSH terms/MeSH subheadings in each period as shown in **Figures 2**–**4**.

In the first period of 2004–2008, Cluster 0 and Cluster 1 were located in Quadrant I. Cluster 0 represents the correlation between brain structural abnormalities and brain function changes to explore physiopathology mechanism of schizophrenia, while Cluster 1 represented neurodevelopmental abnormalities in patients with schizophrenia and analysis of regional abnormal structural features and influencing factors in the brain. These two clusters were developed and in the core status with adequate centrality and high density. Cluster 2 in Quadrant III represented analysis of metabolism, therapeutic efficacy of antipsychotic agents on brain structure and schizophrenia pathology, which had not matured and were in the beginning stages of research in this field.

Compared with the results of 2004–2008, research on brain structure and function in the 2009–2013 period was still located in Quadrant I and was regarded as a mature and developed research area. However, in contrast to the similar theme cluster contents in the previous period, research in the 2009–2013 period was not only focused on the study of brain structure and function in patients with schizophrenia, but also paid more attention to the differential diagnosis of patients with bipolar disorder or other mental disorders. Furthermore, research on the physiopathology mechanisms of schizophrenia and the etiology of cognitive disorder and other complications, the structure and function of the brain default mode network, as well as schizophrenic psychology were newly developed themes.

In the third period of 2014–2018, theme clusters in Quadrant III, including research on differences in the neurobiological basis between schizophrenia and other mental diseases, were similar to the developed and mature theme contents in the first and second periods, which were identified as peripheral and undeveloped themes in the most recent 5 years. In addition, new emerging theme clusters researching on brain structural and functional abnormality of patients with schizophrenia, as well as physiopathology mechanism of the disease were located in Quadrant II, indicating they were developed but still peripheral research areas. Other emerging theme clusters, including effects of genetic load on brain function of schizophrenia patients, as well as identification of schizophrenia biomarkers, were new main undeveloped themes situated in Quadrant IV, which represented central and undeveloped research topics.

To summarize, these three strategic diagrams clearly revealed the current situation and development tendency of each theme cluster of MRI studies on schizophrenia during three different periods.

### Knowledge Structure of MRI Studies on Schizophrenia

**Figure 6:**
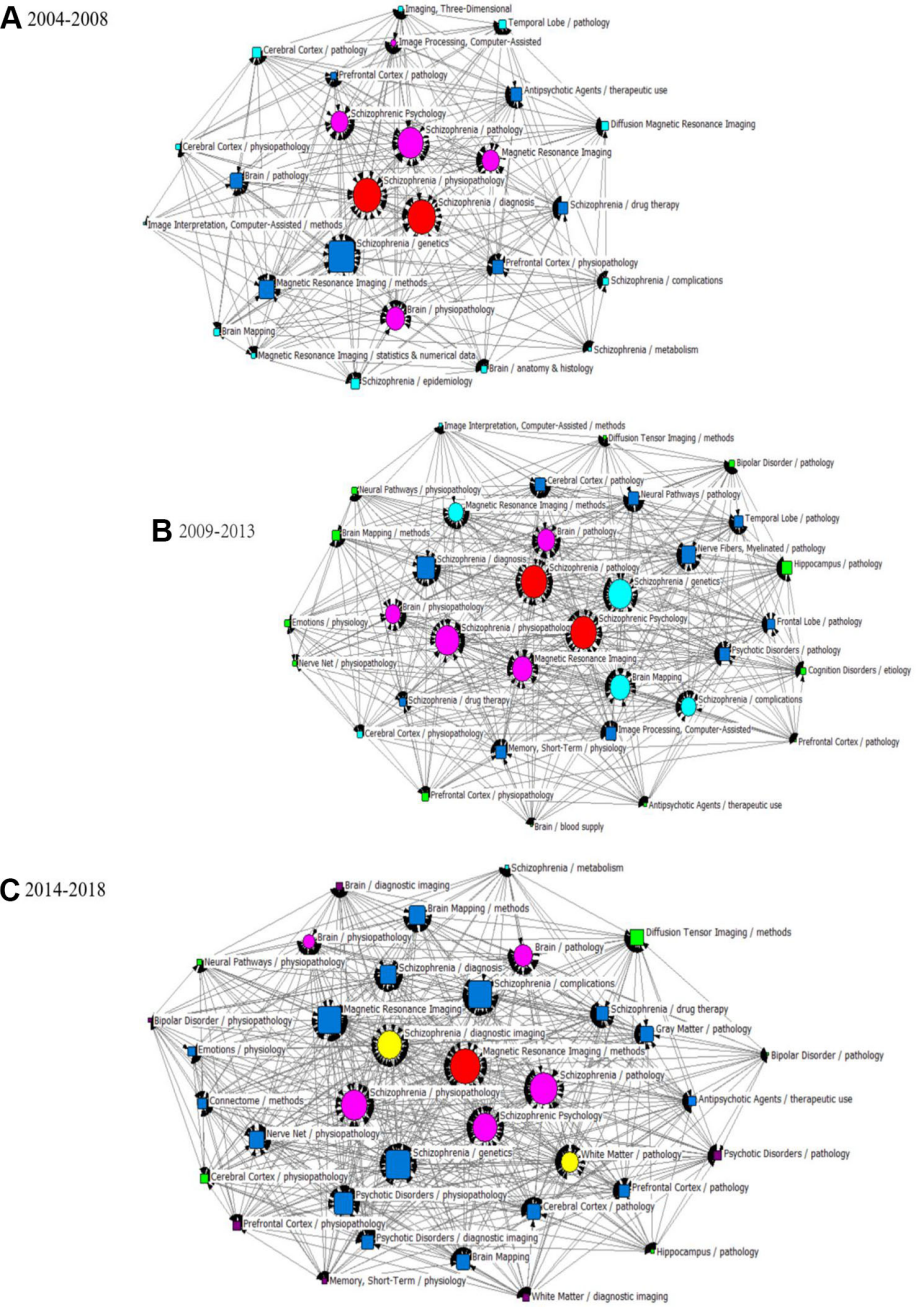
SNA for high-frequency major MeSH terms/MeSH subheadings applied to MRI studies on schizophrenia. **(A)** SNA for 26 high-frequency major MeSH terms/MeSH subheadings in 2004–2008. **(B)** SNA for 34 high-frequency major MeSH terms/MeSH subheadings in 2009–2013. **(C)** SNA for 36 high-frequency major MeSH terms/MeSH subheadings in 2014–2018. The size of the nodes and the thickness of the lines within the networks represent centrality of MeSH terms and the co-occurrence frequency of MeSH terms pairs, respectively. *Red icons (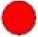):* Node with the highest betweenness centrality. *Circle icons:* Nodes highly co-occurred with other nodes since the period of 2004–2008 (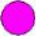), 2009–2013 (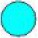) and 2014–2018 (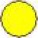), respectively (**Figure 6A–C**). *Box icons:* Emerging nodes in 2004–2008 (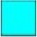), 2009–2013 (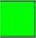) and 2014–2018(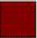), respectively.

In this study, statistical indices of degree, betweenness and closeness centrality were applied to describe the knowledge structure of SNA networks in three different periods (**Tables 4** and **5**). The three SNAs were plotted on the basis of betweenness centrality to gain insight into the results (**Figure 6**). As we commented in the figure legends, the size of nodes was proportional to the betweenness centrality of the major MeSH terms/MeSH subheadings, and the thickness of the lines represents the term-term co-occurrence frequency.

**Table 4:** Descriptive statistics for centrality measures of MRI studies on schizophrenia from 2004 to 2018.

Period	Density	Degree				Closeness				Betweenness			
		Max	Min	$\bar{x}$± S	Network centralization	Max	Min	$\bar{x}$± S	Network centralization	Max	Min	$\bar{x}$± S	Network centralization
2004–2008	8.145±15.599	674.00	41.00	203.69 ± 184.44	17.88%	100.00	62.50	79.90 ± 10.10	42.69%	8.42	0.48	3.39 ± 2.38	1.74%
2009–2013	8.080 ± 17.782	1311.00	73.00	266.65 ± 273.88	15.80%	100.00	66.00	80.05 ± 9.82	41.76%	10.61	0.61	4.41 ± 2.95	1.21%
2014-2018	7.062 ± 16.209	1204.00	48.00	247.17 ± 241.62	13.47%	97.22	64.82	81.18 ± 8.76	33.49%	9.38	0.89	4.31 ± 2.33	0.88%

**Table 5:** Descriptive statistics for centrality measure about MRI studies on schizophrenia from 2004 to 2018.

Period	Rank of MeSH terms	High-frequency MeSH terms/MeSH subheadings	Centrality			Rank of MeSH terms	High-frequency MeSH terms/MeSH subheadings	Centrality		
			Degree	Betweenness	Closeness			Degree	Betweenness	Closeness
2004–2008	3	Magnetic Resonance Imaging	674.000	5.320	89.286	15	Antipsychotic Agents/therapeutic use	99.000	3.174	80.645
	1	Schizophrenia/physiopathology	663.000	8.417	100.000	16	Cerebral Cortex/pathology	93.000	2.587	75.758
	4	Schizophrenia/diagnosis	461.000	8.417	100.000	14	Brain Mapping	91.000	1.650	71.429
	5	Schizophrenic Psychology	429.000	5.313	92.593	21	Diffusion Magnetic Resonance Imaging	86.000	1.927	71.429
	2	Schizophrenia/pathology	415.000	7.593	96.154	20	Brain/anatomy & histology	81.000	1.653	73.529
	7	Image Processing, Computer-Assisted	389.000	1.736	78.125	24	Image Interpretation, Computer-Assisted/methods	72.000	0.482	62.500
	6	Brain/pathology	299.000	3.682	80.645	25	Temporal Lobe/pathology	69.000	2.238	73.529
	8	Brain/physiopathology	275.000	5.543	86.207	11	Schizophrenia/complications	69.000	1.530	71.429
	9	Schizophrenia/genetics	178.000	7.441	96.154	23	Cerebral Cortex/physiopathology	67.000	1.454	71.429
	17	Imaging, Three-Dimensional	162.000	1.246	73.529	19	Magnetic Resonance Imaging/statistics & numerical data	65.000	1.313	71.429
	12	Prefrontal Cortex/physiopathology	144.000	3.388	80.645	26	Prefrontal Cortex/pathology	63.000	1.480	75.758
	10	Magnetic Resonance Imaging/methods	135.000	4.448	83.333	22	Schizophrenia/epidemiology	55.000	2.312	73.529
	13	Schizophrenia/drug therapy	121.000	2.806	80.645	18	Schizophrenia/metabolism	41.000	0.851	67.568
2009– 2013	1	Schizophrenia/pathology	1322.000	10.606	100.000	16	Psychotic Disorders/pathology	140.000	4.133	80.488
	2	Schizophrenia/physiopathology	909.000	9.658	97.059	27	Neural Pathways/pathology	127.000	4.210	80.488
	4	Schizophrenic Psychology	754.000	10.606	100.000	22	Temporal Lobe/pathology	114.000	3.411	78.571
	3	Brain/pathology	700.000	7.333	91.667	25	Neural Pathways/physiopathology	114.000	2.137	71.739
	5	Brain/physiopathology	488.000	5.934	84.615	23	Bipolar Disorder/pathology	114.000	1.953	71.739
	7	Magnetic Resonance Imaging	447.000	8.232	91.667	28	Cognition Disorders/etiology	113.000	2.103	71.739
	6	Brain Mapping	397.000	8.011	91.667	21	Cerebral Cortex/physiopathology	111.000	2.063	71.739
	10	Schizophrenia/complications	320.000	6.084	84.615	26	Brain/blood supply	111.000	0.794	67.347
	9	Magnetic Resonance Imaging/methods	302.000	6.204	84.615	20	Hippocampus/pathology	104.000	3.953	78.571
	8	Schizophrenia/genetics	281.000	9.526	97.059	29	Emotions/physiology	102.000	1.975	71.739
	12	Schizophrenia/diagnosis	246.000	7.139	89.189	34	Image Interpretation, Computer-Assisted/methods	100.000	1.012	67.347
	11	Cerebral Cortex/pathology	244.000	4.293	82.500	30	Brain Mapping/methods	98.000	2.873	75.000
	19	Image Processing, Computer-Assisted	204.000	3.886	78.571	24	Prefrontal Cortex/pathology	94.000	0.607	66.000
	13	Nerve Fibers, Myelinated/pathology	200.000	5.405	84.615	15	Schizophrenia/drug therapy	91.000	2.404	75.000
	17	Nerve Net/physiopathology	172.000	1.468	70.213	31	Frontal Lobe/pathology	86.000	3.229	76.744
	14	Prefrontal Cortex/physiopathology	155.000	2.383	71.739	33	Diffusion Tensor Imaging/methods	85.000	0.917	68.750
	18	Memory, Short-Term/physiology	148.000	4.309	78.571	32	Antipsychotic Agents/therapeutic use	73.000	1.154	70.213
2014- 2018	1	Schizophrenia/physiopathology	1240.000	7.924	97.222	13	Schizophrenia/complications	166.000	7.320	89.744
	2	Schizophrenia/pathology	945.000	8.434	97.222	21	Cerebral Cortex/pathology	155.000	4.272	79.545
	4	Brain/physiopathology	577.000	3.869	83.333	16	Schizophrenia/drug therapy	154.000	3.862	81.395
	3	Schizophrenia/diagnostic imaging	514.000	7.642	94.595	24	Bipolar Disorder/pathology	131.000	0.889	64.815
	5	Schizophrenic Psychology	466.000	7.722	94.595	23	Brain Mapping/methods	129.000	4.990	83.333
	6	Brain/pathology	407.000	5.881	85.366	17	Schizophrenia/diagnosis	126.000	5.120	83.333
	7	Magnetic Resonance Imaging/methods	360.000	9.382	97.222	34	Bipolar Disorder/physiopathology	117.000	1.391	70.000
	9	Brain/diagnostic imaging	279.000	2.225	76.087	27	Memory, Short-Term/physiology	113.000	1.535	71.429
	10	White Matter/pathology	262.000	5.741	87.500	25	Brain Mapping	111.000	4.194	79.545
	14	Nerve Net/physiopathology	240.000	4.592	83.333	30	Psychotic Disorders/diagnostic imaging	111.000	3.946	81.395
	15	Psychotic Disorders/physiopathology	237.000	5.823	89.744	28	White Matter/diagnostic imaging	108.000	2.059	72.917
	8	Schizophrenia/genetics	213.000	7.621	92.105	29	Diffusion Tensor Imaging/methods	107.000	4.112	74.468
	11	Magnetic Resonance Imaging	205.000	7.086	89.744	26	Antipsychotic Agents/therapeutic use	101.000	2.463	72.917
	12	Prefrontal Cortex/physiopathology	198.000	2.618	76.087	32	Neural Pathways/physiopathology	99.000	1.663	72.917
	19	Connectome/methods	194.000	3.040	79.545	31	Emotions/physiology	93.000	2.463	76.087
	18	Cerebral Cortex/physiopathology	182.000	2.482	77.778	35	Prefrontal Cortex/pathology	88.000	3.268	76.087
	22	Psychotic Disorders/pathology	176.000	2.386	72.917	33	Hippocampus/pathology	78.000	1.446	70.000
	20	Gray Matter/pathology	168.000	4.280	79.545	36	Schizophrenia/metabolism	48.000	1.257	68.627

“Rank of MeSH terms” represents the number of high-frequency major MeSH terms/MeSH subheadings in each period as shown in **Figures 2**–**4**.

In the period of 2004–2008, eight major MeSH terms/MeSH subheadings were shown to have a high degree centrality (greater than the mean value of 203.69, **Table 4**) within the network of MRI studies on schizophrenia. As can be seen in **Table 5**, Magnetic Resonance Imaging (674.000) and Schizophrenia/physiopathology (663.000) had higher degree centrality among the eight high-frequency major MeSH term/MeSH subheadings and showed a larger gap between the others. Furthermore, **Table 4** also shows that the top two betweenness centrality values listed in **Table 5** are the same (8.417), which meant that the two major MeSH terms/MeSH subheadings (Schizophrenia/physiopathology, Schizophrenia/diagnosis) had the strongest mediating effect within the network of the first period. Additionally, both terms had the highest closeness values of 100.000, indicating that they had a tight connection with the other nodes. As shown in **Table 5**, another seven major MeSH terms/MeSH subheadings had high betweenness values (greater than the mean value of 3.39, **Table 4**), which suggested that these MeSH terms also played a critical mediating role within the network. In addition, according to **Figure 6A**, the total number of new emerging hotspots in this period was 12, including Magnetic Resonance Imaging/statistics & numerical data, Schizophrenia/epidemiology, Brain Mapping, Brain/anatomy & histology, Schizophrenia/complications, Schizophrenia/metabolism, Diffusion Magnetic Resonance Imaging, Imaging, Three-Dimensional, Cerebral Cortex/pathology, Imaging Interpretation, Computer-Assisted/methods, as well as Cerebral Cortex/physiopathology, which were located on the edge of the network.

Compared with the SNA of the first period, another four new major MeSH terms/MeSH subheadings (**Table 5** and **Figure 6B**), including Brain Mapping, Schizophrenia/complications, Magnetic Resonance Imaging/methods and Schizophrenia/genetics were added to the nodes with high degree centrality in the second period of 2009–2013. Meanwhile, in addition to Brain Mapping and Schizophrenia/complications, another two MeSH terms (Nerve Net/physiopathology and Memory, Short-Term/physiology) were added to the nodes with high betweenness centrality (greater than the mean value of 4.41, **Tables 4** and **5**). Furthermore, the MeSH terms Schizophrenia/pathology and Schizophrenic Psychology had the highest betweenness centrality values (10.606) and the highest closeness centrality values (100.00) (**Table 5**). According to **Figure 6B**, another 12 new emerging nodes were located at the edge of the network, including Brain Mapping/methods, Neural Pathways/physiopathology, Emotions/physiology, Nerve Net/physiopathology, Prefrontal Cortex/physiopathology, Antipsychotic Agents/therapeutic use, Brain/blood supply, Prefrontal Cortex/pathology, Cognition Disorders/etiology, Hippocampus/pathology, Bipolar Disorder/pathology, and Diffusion Tensor Imaging/methods, identifying them as emerging hotspots of MRI studies on schizophrenia in 2009–2013.

In the SNA of 2014–2018, five new major MeSH terms/MeSH subheadings were added to the nodes (**Figure 6C**), including Schizophrenia/diagnostic imaging, White Matter/pathology, Nerve Net/physiopathology, Psychotic Disorders/physiopathology, Brain Mapping/methods, which were characterized by high betweenness values (greater than the mean value of 4.31, **Table 4**). Compared with the SNA of 2009–2013 (**Figure 6B**), a total of six new emerging nodes, including Brain/diagnostic imaging, Bipolar Disorder/physiopathology, Memory, Short-Term/physiology, Psychotic Disorder/pathology, Prefrontal Cortex/physiopathology, and White Matter/diagnostic imaging, were considered as emerging hotspots of MRI studies on schizophrenia in the third period of 2014–2018 (**Figure 6C**).

## Discussion

The human brain is a complex, efficient, and organic system with nearly 100 billion neurons, but during its process of development, a range of genetic, infectious, stress, family, and social environment factors can cause structural and functional abnormalities (34). Schizophrenia has been recognized as the most important brain degeneration disease treated by psychiatrists. Neuroimaging studies have shown that mental disorders can lead to structural and functional changes in brain regions (35, 36), and the structural changes of brain regions are also important risk factors affecting the prodromal stage of schizophrenia (37, 38). The present study evaluated MRI studies on schizophrenia in recent decades by exploring bibliometrics to reach the conclusion that publication of MRI studies on schizophrenia showed an increasing trend in the last 15 years. However, after in-depth analysis, we observed that growth of the third period became slower than the second, but the number of papers published in 2015 is the largest, showing the peak in three curves. In addition, based on the results of the strategic diagram, we observed that contents of theme clusters in each quadrant have changed to some extend so that we could have a preliminary understanding of the theme trends from 2004 to 2018.

In the first period (2004–2008), theme Cluster 0 and Cluster 1 were located in Quadrant I, representing well-developed and mature topics. From the literature analysis, we found that previous research clarified the relationship between brain structural changes and functional abnormalities and analyzed the structural deficit in the corticothalamic system (39), anatomical changes in the frontal-temporal circuit and functional alterations in the prefrontal cortex (40) for the purpose of exploring the etiology and physiopathology of schizophrenia. Furthermore, the structural parts of abnormal brain regions that led to dysfunction and perceived sources of information were identified and positioned (41).

Another theme (Cluster 2) located in Quadrant I. indicated that research on the analysis of metabolism, therapeutic efficacy of antipsychotic agents on brain structure and schizophrenia pathology was immature and needed further study. Previous reports had suggested that after controlling for confounding factors such as sex and age, antipsychotics could alleviate or treat clinical symptoms by altering brain structure (42, 43), but more recent research has found that first-generation antipsychotic drugs may increase oxidative stress and damage cerebral function (44); therefore, whether antipsychotic agents have a protective or destructive effect on brain structure has not been consistently shown. Moreover, numerous studies have demonstrated that almost all cortical and subcortical structures in schizophrenia patients showed morphological abnormalities, including changes in the volume of brain structures such as the whole brain, temporal lobe, amygdala and hippocampus (45–47). Meantime, Fornito, et al. also suggested that impaired brain network connectivity is the pathophysiological basis for diverse clinical symptoms and cognitive impairment of schizophrenia (48). As a result, it is difficult to identify which parts of a brain region that are structurally and functionally abnormal directly contribute to schizophrenia. Therefore, it will be a long process and difficult project to explore the pathological mechanisms of schizophrenia through MRI studies.

During the second period of 2009–2013, partial theme contents of Cluster 1 (research on brain structure and function) were still located in Quadrant I, indicating that researchers kept on paying close attention to the related research topic. With the exception of in-depth studies on nerve fibers, hippocampus, and cerebral cortex (49, 50), good progress has been made in the use of brain imaging to distinguish between schizophrenia and bipolar disorder (51, 52). Moreover, researchers have acquired better understanding of the use of various MRI techniques, their analysis, and interpretation of results (53, 54). In addition, we determined that research on the pathological mechanism of schizophrenia (partial theme contents of Cluster 0, located in Quadrant III) has not yet been fully elucidated and remains as an undeveloped theme. As for the new theme cluster schizophrenia psychology, researchers are dedicated to identifying the basis for the structural and functional activities of brains that cause abnormal psychological symptoms (e.g., emotional withdrawal) in patients with schizophrenia (55, 56). However, due to the diversity of abnormal psychological symptoms in patients and the complicated influencing factors (e.g., course of disease and medication), this field needs further development. Another new theme cluster is brain default mode network (DMN), in which brain network studies have shown changes in the default network functional connectivity in schizophrenia. While results in this area are inconsistent, most studies have shown increased DMN functional connectivity in schizophrenia, as well as weaken functional connections in the prefrontal cortex (57, 58). Nevertheless, taking antipsychotics and changes in psychiatric symptoms can influence resting brain networks, and the intrinsic relationship between genes and DMN have gradually become noteworthy academic issues in recent years (59, 60).

In the third period of 2014–2018, Cluster 1, whose theme contents involve research on differences in the neurobiological basis of schizophrenia and other mental diseases and include studies of the effects of antipsychotics on brain structural changes, was located in Quadrant III. The theme of differential diagnosis of schizophrenia and other mental disorder (e.g., bipolar disorder, schizoaffective disorder), which was originally in Quadrant Iin the second period (2009–2013), is now situated in Quadrant III during the third period (2014-2018). Based on MRI techniques, researchers have studied the similarities and differences in brain structure among patients with schizophrenia and bipolar disorder, such as the two major psychotic disorder have a shared white-matter dysconnectivity in callosal, paralimbic, and fronto-occipital regions (61), but volume reductions in several brain regions of schizophrenic patients, such as the medial frontal cortex, parts of the temporal lobe cortex, the insula and hippocampus cannot be observed in bipolar patients (62). And they also have identified the risk alleles associated with the disease to facilitate more accurate identification and diagnosis (63). Relevant research needs to be further developed and improved.

In addition, as mentioned above, reports about the use of antipsychotics to prevent the progression of brain damage are as yet inconsistent. Numerous studies have found that changes in brain imaging occurred at an early stage in schizophrenia, while structural and functional abnormalities in brain regions caused by grey and white matter volume reductions and anomalous connections, became more prominent with the progression of the condition. But Emsley et al. argued that the brain volume reductions are not directly associated with antipsychotic treatment during the first year of medication, and decrease in brain volume may have more to do with neurotoxicity of drugs than with the therapeutic effect (64). However, due to the differences in research facilities and measuring methods, conclusions are still controversial because abnormal brain regions in various studies do not completely overlap. It remains to be seen whether our next study should take steps to repair brain damage, or whether it should delay, prevent, or interfere with the continued damage to brain structure caused by antipsychotics.

Finally, we can also conclude that the MeSH terms Schizophrenia/physiopathology, Schizophrenia/pathology, and Schizophrenia Psychology have higher or the highest values of betweenness centrality, implying that they have the largest number of direct connections with other nodes, as well as being situated at the core position within the networks. In other words, research on the pathological and physio-pathological mechanisms of schizophrenia, as well as analysis of abnormal psychological and behavioral symptoms from the perspective of brain imaging, are significant and potentially important academic issues in this research field. In addition, the new emerging hotspots in these three periods should be viewed as a guide to finding new directions for research.

## Conclusion

The main finding of this study was that MRI studies on schizophrenia have been an issue of general concern, but the progress (e.g. pathogenesis of schizophrenia) is relatively slow in recent years. Further research is necessary to investigate the undeveloped, immature, and emerging hotspots and theme clusters mentioned above so as to provide medical staff, scientific researchers, and frontline educators with new directions in the field of MRI studies on schizophrenia.

## Limitation

There are several limitations to consider in the current study. To better present results, years were selected as the time unit to divide time periods and complete statistics. However, the complete data of 2019 has not yet been obtained, so the literature published in this year was not included. In addition, this study only included journals articles, so some research hotspots might have been omitted due to the exclusion of conference papers, reviews, and other types of literature. In addition, there are certain limitations in the scope of the PubMed database collection and in the indexing of MeSH terms, so that literature retrieval based on MeSH terms may miss some publications. Furthermore, this study selected the index of major MeSH terms/MeSH subheadings to extract high-frequency MeSH terms, and we cannot completely exclude the possibility that other low-frequency MeSH terms may become hotspots of research in the future. Finally, the data source only selects the published literatures, which may lead to publication bias.

## Author Contributions

GZ designed and corrected the paper. LD wrote the paper.

## Funding

This work was supported by grants from the Major Project of the Department of Science & Technology of Liaoning Province (2019JH8/10300019).

## Conflict of Interest

The authors declare that the research was conducted in the absence of any commercial or financial relationships that could be construed as a potential conflict of interest.
